# 

**Published:** 1883-10

**Authors:** 


ORIGINAL COMMUNICATIONS:

An Examination of the Condition of the Teeth of Certain Pre-Historic American Races. Dr. W. C. Barrett.......... 513
The Use of the Screw in Regulating Teeth. Wm. H. Trueman....... 521
The Curse of Modern Civilization. Dr. George C. Brown........... 526
Dental Periostitis—The Grindstone Cure. Jos. Requa............... 529
The British Dental Association .................. 532

ORIGINAL TRANSLATIONS AND ABSTRACTS:

Minute Anatomy of the Human Tooth.................. 535
Composition of Different Amalgams.................. 541
Kidney Trouble tbe Cause of Toothache.................. 541
German Prize for Dentists' Exhibits.................. 542
Preservative Fluid.................. 542
Hard Tempered Drill.................. 542

REPORTS OF SOCIETY MEETINGS:

New Jersey State Dental Society.................. 542
Report of the Meeting of the Pennsylvania State Dental Society....... 552

EDITORIAL:

Diseases of Pre-Historic Man.................. 559
Erroneous.................. 562
Attention.................. 562

NEW APPLIANCES AND MATERIALS:

The Oxford Tooth Co.................. 562
New Disk Carrier.................. 563
Dental Plaster.................. 563
New Bur and Drill Gauge.................. 464

OBITUARY:

Thomas Lea Buckingham, M. D., D. D. S.................. 564

CURRENT NEWS AND OPINION:

No Time for Reading.................. 566
One Journal Only.................. 567
Preponderance in Sexes.................. 567
Dental Surgery.................. 567
Salivary Calculus.................. 568
American Academy of Medicine.................. 568
Inodorous Iodoform.................. 568
Dental Meeting.................. 568

SELECTIONS:

Notes Upon a Method of Root-Filling.................. 569
The Use of Tooth Brushes.................. 570
Celluloid.................. 571
Hyperidrosis.................. 572
Cincinnati.................. 572

